# Clinical efficacy and pharmacokinetics of meloxicam in Mediterranean buffalo calves (*Bubalus bubalis*)

**DOI:** 10.1371/journal.pone.0187252

**Published:** 2017-10-27

**Authors:** Petra Cagnardi, Jacopo Guccione, Roberto Villa, Luigi D’Andrea, Antonio Di Loria, Maria Carmela Ferrante, Giuliano Borriello, Luigi Zicarelli, Paolo Ciaramella

**Affiliations:** 1 Department of Health, Animal Science and Food Safety – Università degli Studi di Milano, Milano, Italy; 2 Department of Veterinary Medicine and Animal Productions - University of studies of Napoli “Federico II”, Napoli, Italy; 3 Department of Experimental and Clinical Medicine - University of Magna Graecia of Catanzaro, Catanzaro, Italy; University of Bari, ITALY

## Abstract

The aims of the investigation were to establish for the first time (i) clinical efficacy and (ii) pharmacokinetic profile of meloxicam intravenously (IV) administered in male Mediterranean buffalo calves after surgical orchiectomy. The study was performed on 10 healthy buffalo calves, between 4 and 5 months old and between 127 and 135 kg of body weight (b.w.). An IV injection of 0.5 mg/kg b.w. of meloxicam was administered in six calves (treated group, TG) immediately after surgery; the other four animals were used as untreated control group (CG). The clinical efficacy of meloxicam was evaluated pre- and post-surgery by monitoring respiratory rate (RR), heart rate (HR), rectal temperature (T°C), serum cortisol levels (SCL) and pain score (PS). Significant inter-groups differences were detected at sampling times (T): 4 hour (h) for RR (*P<*0.05), at T1-4-6-8 h for PS (*P<*0.05) and at T4-6-8 h for SCL (*P* < 0.0001). Regarding the mean intra-group values observed pre (T0) and post-surgery (from T15 min to T72 h), significant difference between the groups were found for RR (*P<*0.01), PS and SCL (*P<*0.05). The pharmacokinetic profile was best fitted by a two-compartmental model and characterized by a fast distribution half-life and slow elimination half-life (0.09 ± 0.06 h and 21.51 ± 6.4 h, respectively) and meloxicam mean concentrations at 96 h was of 0.18 ± 0.14 μg/mL. The volume of distribution and clearance values were quite low, but reasonably homogenous among individuals (V_dss_ 142.31 ± 55.08 mL/kg and Cl_B_ 4.38 ± 0.95 mL/kg/h, respectively). The IV administration of meloxicam in buffalo calves shows encouraging effects represented by significant and prolonged analgesic effects, significant reduction of SCL as well as similar pharmacokinetic profile to bovine calves.

## Introduction

The European population of domestic buffalo (*Bubalus bubalis*) counts of approximately 395.000 heads [1], and Italy represents, with his 369.352 heads of Mediterranean buffalo, the main rearing country (95%) [2]. Primarily characterized by mozzarella cheese production [2], buffalo’s breeding in Italy, recently has also recorded a significant growing of the beef buffalo’s market [3,4]. As widely described for bovine [5,6], in order to reduce aggression, sexual activity, and incidence of dark-cutting carcasses, meat production often forces veterinary practitioners to castration of young buffalo male calves. While in bovine, during minor surgical procedures, the pain and welfare management are well-established on practice for their ethical implications and impact on productivity [7], on buffalo these aspects, as well as clinical related procedures, are still truly rare [8,9].

In general, observation of behavioural changes (e.g. abnormal standing-time, lying-time or posture, reduced walking time) associated with clinical triage monitoring (i.e. respiratory-rate, hearth-rate and temperature) are common parameters evaluated by the veterinary practitioners in field, although their accuracy to reflect pain perception is recognised as low [9,10,11]. On the other hand, the evaluation of some haemato-biochemical parameters is instead recommended as the most reliable method to evaluated welfare in animals [7,12] and serum cortisol level can be considered as the analytic, neuroendocrine parameter indicative of animal stress, due to the direct influence on the corticosteroids release in the blood. Indeed, it is widely proved that serum cortisol concentration tends to rise when an increased activity from nociceptor is elicited by a pain stimulus [13].

As reported in literature, pain perception in cows can be significantly reduced by administration of non-steroidal anti-inflammatory drugs (NSAIDs) often associated to the most common anaesthetic protocols during several minor and major surgical procedures [7]. NSAIDs exert anti-inflammatory action through variable inhibition of the cyclooxygenase iso-enzymes (COX-1 and -2). Meloxicam belongs to this category of drugs and can be considered a preferential inhibitor of COX-2: the isoform greatly up-regulated in presence of inflammatory stimuli [14,15]. Since a long time, meloxicam properties and effects were investigated in several domestic species including dogs [16], cats [17], camels [18] and cows [7,19,20,21]. Its use is approved for cattle in EU-countries, as well as in several non-EU-countries (e.g. Canada, New Zealand, Australia); it is often recommended for endotoxicosis [22] and mastitis treatments [23] or pain relief after several surgical procedures in cows and calves [20,21,24]. In the latters, the administration of meloxicam at 0.5 mg/kg IV or intramuscular (IM) decreases behavioural and physiological responses to pain and distress associated with dehorning [25,26,27]. Moreover, its pharmacokinetic profile and its effects on behaviour and performance suggest that these may last for several days after administration [21]. In young and adult cattle, meloxicam demonstrated a high oral bioavailability and long elimination half-life, providing an effective and long-lasting analgesia [28]. At present, meloxicam is commonly used in buffalo practice, although no studies about its clinical efficacy, pharmacokinetics or pharmacodynamics have been published so far.

Considering the premises, the goals of the present investigation were to establish (i) clinical efficacy and (ii) pharmacokinetic profile of meloxicam IV administered in male buffalo calves after surgical orchiectomy.

## Materials and methods

### Animals

Ten male buffalo calves, 3 and 4 months of age (median = 3.65 months), 127 and 135 kg of body weight (median = 130 kg), reared in the same breeding farm in Caserta district (Southern Italy), were enrolled for this study.

The animals were randomly chosen from a group of 35 buffalo male calves and were individually submitted to a complete clinical examination and blood samples for routine haemato-biochemical investigations (data non shown) to verify the general good health status. One week before the orchiectomy, 4 calves were chosen as negative control group among the 10 selected animals (CG, untreated with meloxicam after castration), using a random number-generated software package (Microsoft Excel); the remaining 6 buffaloes were identified as treated group (TG, treated with meloxicam after castration). Both the groups (TG and CG), were place in one, roofed, common paddock of ~ 50 m^2^ (~ 10 m × 5 m) and they were fed with a total mixed ratio including hay, silage and a multi-vitamin integrator one time a day; water was provided ad libitum. During the week before the castration, the investigators, to reduce the influence of human presence on animals’ behavior and stress levels, daily handled all the animals. Respiratory rate (RR), hearth rate (HR) and rectal temperature (T°C) were also daily recorded in order to get the calves used to the clinical procedures.

### Drugs administration and surgical procedure

Two animals every 96h were castrated (5 groups of 2 calves). One h before surgery (T0), after trichotomy and surgical scrub of the jugular region [29], all the buffaloes received an IV catheters (14 Gauge, L/A long-term catheter, Anicath, Millipledge, UK), for drug administration and blood samples collection. Catheter was flushed with 10 mL of NaCl 0.9% after each use; calves were IM treated with a 5 mL of oxytetracycline (Oxtra MV 10, Fatro, Ozzano, Italy) after blood collection.

Animals were sedated with 5 μg/kg b.w. of dexmedetomidine IV (DexDomitor, Vétoquinol, Ozzano, Italy); they also received 4.5 mL of Lidocaine 2% (Zoetis Italia, Roma, Italy), injected into each spermatic cord, and further 1 mL in the incision line. Waiting for drugs effects, calves were placed in right lateral recumbency, and surgical field was prepared by scrubbing the scrotal, inguinal and perianal areas according to Desrosches guidelines [29]. Surgical orchiectomy was performed according to the procedure described by Weaver et al. [30]. The surgery was considered concluded after the removal of both testicles and the topic application of oxytetracyclin (Neo spray CAF, Intervet, Milano Italy); then all the calves received 0.05 mg/kg b.w of atipamezole IV (Atipam, Dechra, Northwich, UK) to antagonize the sedative and TG received a single dose of 0.5 mg/kg b.w. of meloxicam IV (Metacam^®^ 20 mg/mL, Boeringher Ingelheim Vetmedica Inc. Ingelheim am Rhein, Germany). Subsequently blood samples were routinely collected two times (5 and 10 min after surgery) to perform venous blood gas analyses (using i-STAT analyser, Abbott, Worcester, US) to monitor the acid-base balance of the patients.

### Clinical procedures

At T0, all the buffalo calves received on additional individual clinical examination to confirm the good health status and to verify the serum cortisol levels (SCL) pre-surgery; moreover, they were scored according to physical status scale of the American Society of Anaesthesiologists to predict operative risk [31]. At the same time, calves were also scored with a unidimensional, composite, pain scale validated for cows [32]. Briefly, the scoring system tracked five behavioral changes (1-locomotion, 2-interactive behaviour, 3-activity, 4-appetite and 5-miscellaneous behaviours) by an ordinal point scale, ranged from 0 (normal) to 2 (totally abnormal), and applied to each parameter, as specified in Table 1. Each subject received 5 minutes (min) of continuous monitoring and could achieve a maximum cumulative pain score (PS) of 10 points. All the scorings were independently and blinded performed by three observers. The observers were placed (one per corner) within the animals’ paddock, approximately 25 min before the evaluation, in order to adapt the calves to their presence.

**Table 1:** Pre- and post- orchiectomy UNESP-Botucatu unidimensional pain scale used at each sampling time.

Parameters	Score/Criterion
**Locomotion**	(0) Walking with no obviously abnormal gait (1) Walking with restriction, ma be with hunched back and/or short steps (2) Reluctant to stand up, standing up with difficulty or not walking
**Interactive behaviour**	(0) Active: attention to tactile and/or visual and/or audible environmental stimuli; when near other animals, can interact with and/or accompany the group (1) Apathetic: may remain close to other animals, but interacts little when stimulated (2) Move less frequently in the pasture or only when stimulated
**Activity**	(0) Moves normally (1) Restless, moves more than normal or lies down and stands up with frequency (2) Move less frequently in the pasture or only when stimulated
**Appetite**	(0) Normorexia and/or rumination (1) Hyporexia (2) Anorexia
**Miscellaneous behaviours**	Wagging the tail abruptly and repeatedly Licking the surgical wound Moves and arches the back when in standing posture Kicking/foot stamping Hind limbs extended caudally when in standing posture Head below the line of spinal column Lying down in ventral recumbency with full or partial extension of one or both hind limbs. Lying down with the head on/close to the ground Extends the neck and body forward when lying in ventral recumbency (0) All of above described behaviours are absent (1) Presence of 1 of the behaviours described above (2) Presence of 2 or more of the behaviours described above

After surgery, clinical efficacy of meloxicam was evaluated on regular base by means of RR, HR, T°C and PS detection. The parameters (RR, HR, T°C) were always assessed by the same 2 investigators (one per animal), at times (T) 15, 30, 45 min and 1, 1.5, 2, 4, 6, 8, 12, 24, 36, 48 and 72 h post-orchiectomy; at the same times, calves received a blood sample collection for SCL evaluation. RR was evaluated observing the chest-abdominal movements on the right side of the calves, HR using a stethoscope (Litmann Master Classic II, 3M Health Care, Minnesota) placed at level of mitral valve and T°C with electronic thermometers (Digi-Vet SC10, Kruuse; Denmark). The same investigator recorded the previous parameters 3 times and the mean value was considered for statistical analysis. Finally, each animal received a further complete clinical exanimation and an additional blood sample was collected to define its health status before the dismissal (T96 h).

All the procedures performed followed the *common good clinical practices* [33] and received an institutional approval by Ethical Animal Care and Use Committee of University of Naples “Federico II” (Protocol No. 67990/2015); moreover the farmer was previously informed and in agreement with purposes and methods used.

### Serum cortisol analysis

All the blood samples were placed into serum tubes (Vacutainer^®^, Becton and Dickinson, Franklin Lakes, US) and centrifuged to obtain the sera (908 *g* × 15 min). Cortisol concentrations were measured on serum, using a solid phase competitive chemiluminescent enzyme immunoassay and an automated analyser system (Immulite 1000 Cortisol, Siemens Medical Solution Diagnostic) according to the procedure described by Coetzee et al. [21]. A sample volume of 100 μL was used for each assay. The calibration range was 28–1380 nmol/L and the analytical sensitivity was 5.5 mmol/L.

### Meloxicam determination

The sera, obtained from all the blood samples performed, were also used to determine the meloxicam concentration and pharmacokinetics. Meloxicam was extracted with a liquid-liquid procedure from sera of TG according to the method described by Gao et al. [34] and validated in the laboratory. The meloxicam serum quantification was performed by HPLC system that included a binary pump, an autosampler, a Peltier column oven set at 20°C and an UV/Visible detector (Series 200, Perkin Elmer, Italy) set at 360 nm of wavelength.

The drug separation was achieved by Zorbax column C18 (150x4.6, 3.5 μm, Agilent Technologies, USA) with adequate pre-column. A gradient HPLC method was used for the analysis. The mobile phase consisted of a mixture of water, 0.5 mL/L phosphoric acid and 0.75 mL/L triethylamine (A) and methanol (B) with a flow rate of 1 mL/min. The HPLC gradient was (A:B, v/v) 35:65 for 7 min; 0:100 in 1 min; 0:100 for 3 min; 30:65 in 1 min and 30:65 for 4 min to re-equilibrate the system.

The analytical standard of meloxicam sodium salt hydrate (purity grade 99%) was provided by Sigma Aldrich (Italy). All reagents and solvents were purchased from Panreac Sa (Italy).

The analytical method was validated intra-laboratory using a set of parameters (linearity, accuracy and precision, limit of quantification, i.e. LOQ, limit of detection, i.e. LOD and selectivity) that were in compliance with the recommendations defined by the European Community [35] and with the international guidelines [36]. The calibration curves were prepared with spiked solutions obtained diluting the original stock solution of meloxicam (1 mg/mL) in buffalo blank serum to achieve concentrations ranging from 0.01 to 10 μg/mL. The correlation coefficients (r) resulted > 0.99 for 3 replicates.

The precision (repeatability) and accuracy were determined by analysing blank samples (n = 6 for each concentration) that were spiked with 0.01, 1 or 10 μg/mL. The results fell within the accepted ranges for precision (8.9%, 11.3% and 4.5% for 0.01 μg/mL, 1 μg/mL and 10 μg/mL, respectively) and accuracy (108.6%, 109.1% and 108.5% for 0.01 μg/mL, 1 μg/mL and 10 μg/mL, respectively). A LOQ value of 0.01 μg/mL was defined. The LOD was 0.045 ng/mL. The specificity of the method was demonstrated by the absence of interference in 20 blank serum samples at the meloxicam retention time.

### Pharmacokinetic analysis

Pharmacokinetic parameters were deduced from serum concentration-time data using software (WinNonLin Prof 6.3, Pharsight Corporation, USA) which allows compartmental and non-compartmental analyses of the experimental data. Visual inspection of the curve, residual analysis and minimum Akaike’s information criterion estimation [37] were used to choose the model best fitting the data. All data points were weighted by the inverse square of the fitted value. The disposition of meloxicam following IV administration was described by a classical two-compartments model [38].

### Statistical analysis

Clinical variables (RR, HR, T°C and PS), SCL and pharmacokinetic parameters were analyzed by standard descriptive statistics; normality was assessed using histograms, normal probability plots and Shapiro Wilk tests. Data were expressed as absolute numbers, percentage, median and range, or mean ± standard deviations (SD). For the pharmacokinetic parameter of half-lives harmonic means with pseudo-standard deviations (SE) were calculated using a jack-knife technique suggested by Lam et al. [39]. Regarding the clinical parameters (RR, HR, T°C and PS) and SCL, differences inter- (TG vs. CG, at each sampling time) and intra- groups (T0 vs. mean value from T15 min to 72 h) were compared using Repeated-Measures Factorial ANOVA and nonparametric Mann-Whitney U test, respectively. Correlation between mean values of the continuous variables (RR, HR, T°C, PS and CS) at each sampling time was also investigated both for TG and for CG by means of non-parametric *Spearman*'s rank correlation test (intra-group). As described by Taylor [40], correlation coefficients (r_s_) of r_s_ ≤ 0.35 were scored as weak, r_s_ between 0.67 and 0.36 as moderate, between 0.89 and 0.68 as high and r_s_ ≥ 0.9 as very high.

Regarding PS, inter-rater agreement between the quantitative measures of the three different observers was also calculated using the intraclass correlation coefficient (ICC) based on a two-factor mixed-effects model and type absolute agreement, according to Montgomery et al. [41].

*P*robabilities < 0.05 were considered as significant. All statistical analyses were performed using dedicated software (SPSS, Version 17.0, Chicago, IL).

## Results

### Clinical parameters and serum cortisol

All the animals submitted to ASA classification were included in the *Class I*. No adverse effects were observed during and after meloxicam administration in the buffalo calves enrolled. All of them presented scrotal oedema due to the surgery procedures, spontaneously recovered within 24–36 h after surgery (median = 28.5 h).

The overall mean RR values recorded were significantly lower (*P <* 0.001) in TG [19.5 ± 4.0, breath per min (bpm) ±SD] than CG (23.7 ± 6.4 bpm). Comparing the two groups at each sampling time, TG showed significant lower values than CG at T4 h after surgery (19.4 ± 3.6 and 27.0 ± 2.4, respectively; *P <* 0.05).

Instead, the mean intra-group values observed pre- (T0) and post-surgery (average from T15 min to T72 h) revealed significant lower values within TG (T0 = 25.7 ± 4.9 bpm vs. mean T15 min-T72 h = 19.0 ± 3.5 bpm; *P <* 0.01) than CG (T0 = 31.0 ± 9.2 bpm vs. mean T15 min-T72 h = 23.1 ± 5.9 bpm). No inter- (overall and partial values) or intra-groups significant differences were instead recorded for HR and T°C.

Regarding the PS, a near perfect agreement between the observers was found (ICC = 0.97, *P*< 0.0001; CI_95_ = 0.94 to 0.98). The mean overall pain scores recorded were significantly lower (*P <* 0.05) in TG (3.5 ± 3.3 points) than CG (4.9 ± 3.5 points). Comparing the two groups at each sampling time, TG showed significant lower values than CG at T1h (TG = 6.8 ± 0.9 vs. CG = 8.0 ± 0.8; *P <* 0.05), T4h (TG = 2.3 ± 0.5 vs. CG = 6.5 ± 1.9; *P <* 0.05), T6h (TG = 1.7 ± 0.7 vs. CG = 6.0 ± 1.6; *P <* 0.05) and T8h (TG = 2.3 ± 0.2 vs. CG = 5.0 ± 1.1; *P <* 0.05) after surgery. A significant difference between the mean intra-group values observed pre- (T0) and post-surgery (average from T15 min to T72 h) were detected both within TG (T0 = 0.6 ± 0.4 points vs. T15 min-T72 h = 3.7 ± 3.3 points; *P* < 0.05) and CG (T0 = 0.3 ± 0.0 points vs. T15 min-T72 h = 5.2 ±3.3 points; *P* < 0.01).

**Fig 1:**
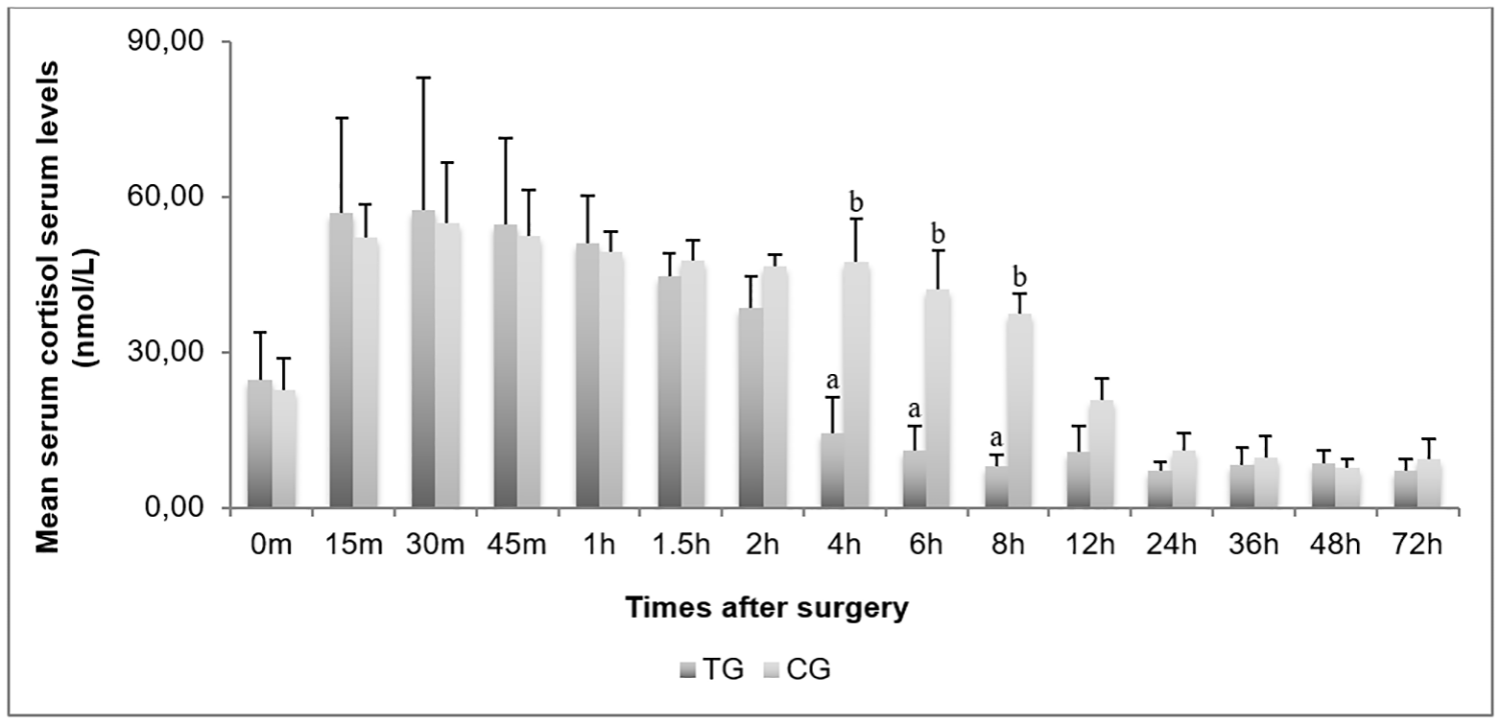
Mean serum cortisol levels pre- (T0) and post- orchiectomy (from T15m to T72h). TG = treated groups; CG = control group; min = minutes; h = hours;T = time; ^a,b^ *P <* 0.0001.

The overall mean SCL values recorded were significantly lower (*P <* 0.01) in TG (26.8 ± 22.6 nmol/L) than CG (34.1 ± 18.6 nmol/L). Comparing the two groups at each sampling time, TG showed significant lower values than CG at T4h (TG = 14.4 ± 7.0 vs. CG = 47.4 ± 8.4; *P* < 0.0001), T6h (TG = 11.0 ± 4.7 vs. CG = 42.2 ± 7.3; *P* < 0.0001) and T8h (TG = 8.0 ± 4.8 vs. CG = 37.4 ±4.0; *P* < 0.0001, Fig 1). The mean intra-group values observed pre- (T0) and post-surgery (average from T15 min to T72 h) were significantly higher in CG (T0 = 22.7 ± 6.0 vs. mean T15 min-T72 h = 34.9 ± 18.9 nmol/L; *P* < 0.05), than in TG (T0 = 24.6 ± 9.2 vs. mean T15 min-T72 h = 27.0 ± 23.3 nmol/L).

Finally, a very high correlation was detected between SCL and PS both in TG (r_s_ = 0.97; *P*< 0.01) and CG (r_s_ = 0.95; *P*< 0.01); instead it was moderate between SCL and T°C in CG (r_s_ = 0.75; *P*< 0.01).

### Meloxicam concentrations and pharmacokinetic analysis

**Fig 2:**
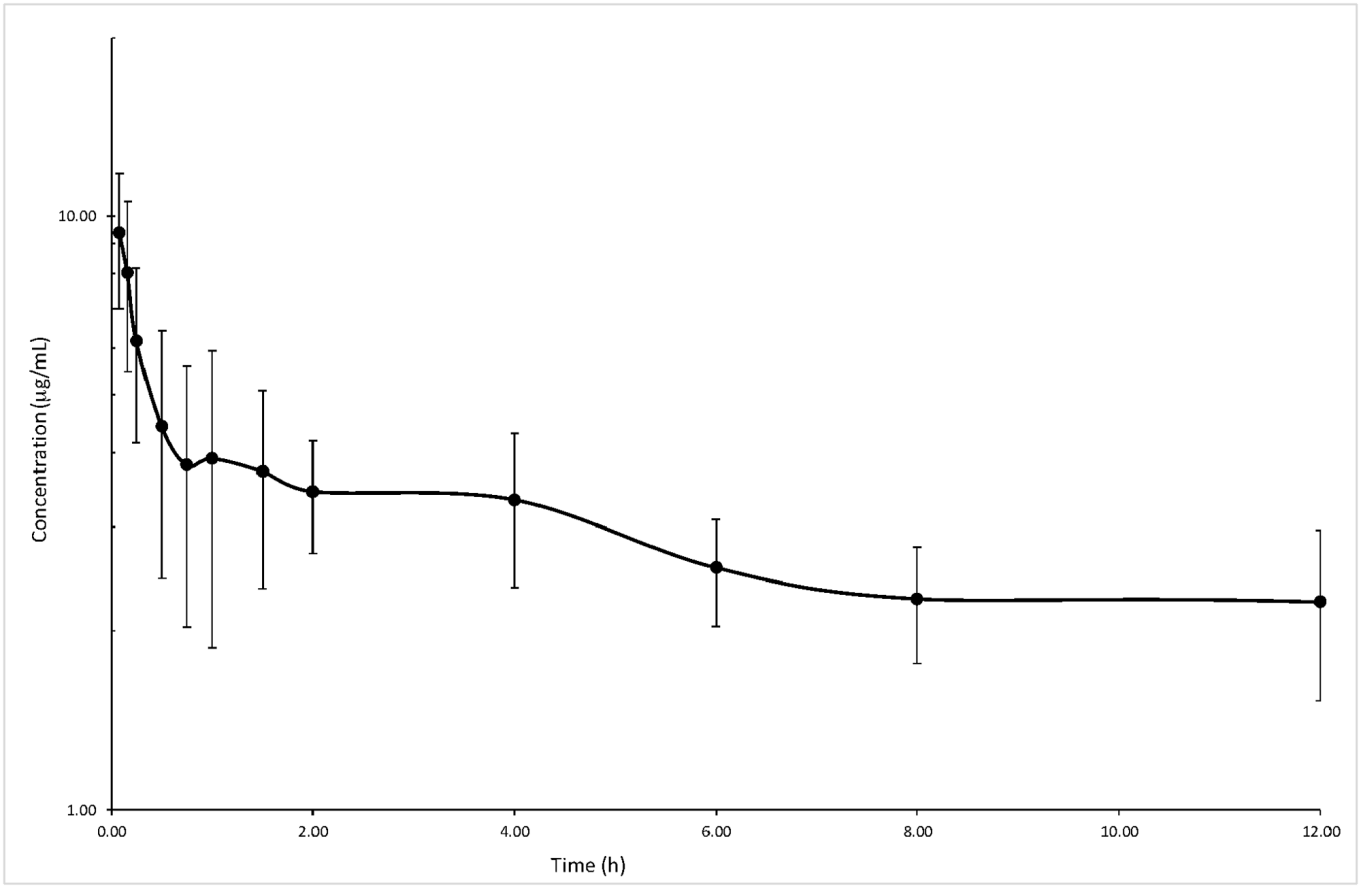
Mean meloxicam concentrations (± S.D.) time profile from 0 to 96 h after IV treatment at 0.5 mg/kg in 6 buffalo calves.

Mean serum concentration of meloxicam in 6 buffaloes is shown in Fig 2. The mean concentration at the first sampling time was 9.39 ± 2.39 μg/mL. Then, there was a decreased in plasma concentration and at 0.75 h mean value was 3.81 ± 1.78 μg/mL. Successively, concentrations remained in a mean range between 3.91–2.24 μg/mL till 12 h after treatment. From 24 to 96 h meloxicam concentration progressively decreased from 1.88 ± 0.53 μg/mL to 0.18 ± 0.14 μg/mL. Results from all subjects were best fitted by a two-compartmental model and Table 2 reports all the results.

**Table 2:** Mean and S.D. of meloxicam pharmacokinetic parameters after IV administration at 0.5 mg/kg in 6 buffalo calves.

Parameters	Unit	Mean	S.D.
AUC _(0→∞)_	h* μg/mL	118.81	27.57
t ½ _K10_	h	6.93	3.66
t ½ _α_	h	0.09*	0.06
t ½ _β_	h	21.51*	6.4
K10	1/h	0.13	0.09
K12	1/h	4.18	2.89
K21	1/h	1.75	1.61
C0	μg/mL	14.39	7.13
V_1_	mL/kg	40.51	15.16
Cl_B_	mL/h/kg	4.38	0.95
AUMC_(0→∞)_	h*h* μg/mL	3991.78	1859.87
MRT_(0→∞)_	h	32.89	10.33
V_dss_	mL/kg	142.31	55.08
V_2_	mL/kg	101.8	67.31
V_z_	mL/kg	146.16	53.98

AUC _(0-∞)_ = area under serum concentration-time curve from 0 extrapolated to infinity; t ½ K10 = the half-life associated with the rate constant K10; t ½α = distribution half-life; t ½β = elimination half-life; K10 = the rate at which the drug leaves the system from the central compartment (the elimination rate); K12 = the rate at which the drug passes from central to peripheral compartment; K21 = the rate at which the drug passes from peripheral to central compartment; C0 = serum concentration at time 0; V1 = volume of distribution in the central compartment; Cl_B_ = body clearance; AUMC = area under moment curve; MRT = mean residence time; V_dss_ = volume of distribution at steady-state; curve; V2 = volume of distribution in the peripheral compartment; V_z_ = volume of distribution based on the terminal phase;

* = harmonic mean ± pseudo-deviation standard.

## Discussion

To the best of the authors’ knowledge, in this paper the clinical efficacy and pharmacokinetics of meloxicam is investigated for the first time in buffalo calves. The legal inclusion of buffalo within the bovine specie by the EU since 2009 [42], promoted the use of drugs manufactured for bovine in these ruminants without scientific proves of their efficacy; for this reason, the authors used a careful and methodical approach for the evaluation of the outcomes of a drug originally produced for dairy cows, as performed in previous studies [43,44].

In cow calves, after minor surgical procedures (e.g. castration, dehorning), meloxicam administration can significantly reduce pain, stress and increase profitability by increasing body average daily gain and reducing susceptibility to disease [20,21]. Meloxicam can be administered by two routes in cattle: oral and parenteral. Although the oral one demonstrated a good applicability, bioavailability and extended effects [19,28], it needs to be administered 12 h before surgery to coincide with the peak of drug concentrations [19]. In our experimental condition, the choice of IV administration was performed both because of the faster positive analgesic effects reported in cow calves and because of the avoidance of any influence in bioavailability and drug distribution, due to other routes of administration [19,21,45].

Pain assessment in ruminants represents a true challenge for clinicians in field. Typically, prey in their natural state, they tend not to express pain to limit vulnerability [32]. Although some behavioural indices to assess the pain perception in cattle have been already developed [7], only the “UNESP-Botucatu unidimensional composite pain scale” has been validated for postsurgical pain assessment in dairy cows so far [32]. Thus, in buffaloes the lack of specific pain score scale has forced us to use the model created for dairy cows. Pain stimulus caused by castration is often associated to physiological, behavioral and neuroendocrine changes [7]. Indeed, after the release of pro-inflammatory cytokines and the consequent activation of the hypothalamus-pituitary-adrenal axis, the sympathetic nervous system bring to the release of ACTH and catecholamines [46]. An increase of the myocardial activity and the peripheral vasoconstriction are the main effects raised by this neuroendocrine activity followed by a higher oxygen request generating an increase of RR and HR, as consequence [46]. Thus, these clinical parameters can be considered an indirect indicator of pain. The significant lower RR values observed in TG than CG may confirm the analgesic effects of meloxicam administrated in buffalo calves post orchiectomy. The result confirms similar findings observed in cow calves after dehorning and reporting significant RR decrease in the treated animals [25].

Unlike a similar study based on intravenous administration of meloxicam in dairy calves during dehorning [21], the HR did not showed any difference between TG and CG. The lack of difference may be due to the different surgical procedure as well as to the technique used for HR detection; although an adaptation phase has been introduced, the handling of the animals, necessary for parameter detection, may have reduced the difference between the two groups. Probably, the use of different recording systems such as digital Holter, already used in farm animals for scientific purposes, may allow a correct detection over-time HR, reducing the negative effects due to investigators presence [47].

Regarding the body temperature, the administration of meloxicam did not produce any significant difference between the two groups confirming similar finding recently observed in beef bull calves submitted to surgical castration [48]. The correct application of the good clinical practices may explain the results observed. As described by Desrosches [29], the respect of high hygiene standards pre-, during- and post-surgical procedures may significantly reduce risk of secondary infection during the follow-up period. Although no significant differences have been observed between the two groups, a moderate correlation between T and SCL have been found only in CG. The difference may be explained considering that the decreasing curve of the values of both the parameters (T and SCL) in this group was likely more similar than those observe in TG were the SCL decrease more quickly.

Finally, the overall significant difference observed between TG and CG regarding the PS seemed to confirm the painkiller properties of the NSAIDs allowing a reduction of pain behaviours. Although the surgical procedure produced a series of behavioural changes in both groups, treated animals were also scored significantly lower than the untreated, when compared with their respective T0 (pre-surgery) further supporting the hypothesis. Moreover, the significant correlations observed between PS and SCL in both the groups may confirm the reliability of this composite pain scale for assessing postoperative pain also in buffaloes.

Concerning the SCL, the parameter showed lower overall mean values in TG than CG, as observed also by Coetzee at al. [21] in animals after dehorning. Moreover in our study, although both groups achieved a SCL peak 30 min post-orchiectomy, the TG showed a faster and significant decrease of SCL (T4 h) than CG in which analogous values were reached only the day after (T 24 h) (Fig 1). The results seem to confirm how meloxicam may have inhibitory effects on the COX iso-enzymes also in buffalo as in cow, reducing the pain perception, subsequent stress with positive effects on the animal welfare; indeed, a significant decrease of SCL after stressors action (e.g. orchiectomy), can protect the animals against its immunosuppressive effects, reducing their long-term susceptibility to infections [23].

The pharmacokinetics of meloxicam after oral and IV administration has been first described by Coetzee at al. 2009 [19] in calves. A mean peak plasma concentration (C_max_) of 3.10 μg/mL (range 2.64–3.79 μg/mL) was recorded at 11.64 h (range 10–12 h) with an elimination half-life of 27.54 h (range 19.97–43.29 h) after oral meloxicam administration at 1 mg/kg. After IV administration at 0.5 mg/kg the elimination half-life and Cl_B_ were 20.35 h (range 17.84–22.76 h) and 6 mL/kg/h (range 4.8–7.2 mL/kg/h), respectively, whereas, V_dss_ and MRT were 171 mL/kg (range 151–189 mL/kg) and 28.19 (range 24.65–31.58), respectively [19].

The pharmacokinetic profile of meloxicam in buffalo was characterized by a fast distribution half-life (0.09 ± 0.06 h) and slow elimination half-life (21.51 ± 6.4 h) and after a single IV injection meloxicam was quantifiable for several days with a mean concentrations of 0.18 ± 0.14 μg/mL at 96 h. Despite the young age of our buffaloes and the presumable variable metabolic capacity, the low V_dss_ and Cl_B_ values were reasonably homogenous among individuals, with values of 142.31 ± 55.08 mL/kg and 4.38 ± 0.95 mL/kg/h, respectively. As reported for cows, the high protein binding of meloxicam (96.5–98%) [49] can justify the limited volume of distribution observed in our buffalo calves; this binding can cause relatively low drugs distribution into the interstitial fluids but facilitates passage into areas of inflammation with leakage of plasma proteins into exudate [50].

The low Cl_B_, is responsible for the quite long elimination half-life and is influenced by the hepatic metabolism, that is necessary for meloxicam elimination from the body [51]. The V_dss_ (142.31 ± 55.08 mL/kg) was very similar to the V_z_ (146.16 ± 53.98 mL/kg). Thus, in our buffaloes minimal amounts of meloxicam were eliminated during the distribution phase.

The pharmacokinetic profile observed in buffaloes was quite similar to that observed more recently in Holstein calves undergoing dehorning by Coetzee et al. [21] after IV administration of 0.5 mg/kg. The distribution half-life was shorter in buffaloes than in calves (0.09 ± 0.08 vs. 0.22± 0.087 h), Cl_B_ was more rapid in Holstein calves (6.64 ± 0.76 vs. 4.38 ± 0.95 mL/h/kg), but elimination half-lives and MRT were comparable (t½_β_ 21.86 ± 3.03 in calves and 21.51 ± 6.4 h in buffaloes; MRT 31.24 ± 4.37 in calves and 32.89 ± 10.33 h in buffaloes). Volumes of distribution were higher in Holstein calves (V_dss_ 193.94 ± 10.34 vs. 142.31 ± 55.08 mL/kg; V_1_ 94.88 ± 9.04 vs. 40.51 ± 15.16 mL/kg), whereas AUC was higher in buffaloes (118.81 ± 27.57 vs. 81.08 ± 10.58 h* μg/kg). The results in buffaloes were comparable also to those first published calves after IV administration at the same dose by Coetzee et al. 2009 [19], thus, the assumption that genetic and physiological differences between buffalo (*Bubalus bubalis*) and cattle (*Bos Taurus)* can have a significant impact on pharmacokinetics [52,53] was not completely confirmed in our study with meloxicam, although this evaluation could have been limited by the reduced number of animals treated (n = 6).

The association of the positive effects as painkiller together with the pharmacokinetic profile of meloxicam indicate that this drug can have effects for several days in buffalo calves. In our study, meloxicam was administered immediately after surgery, but to optimize the perioperative analgesia and in agreement with a pre-empitive analgesia, meloxicam could be administered IV to buffaloes 20–30 min before surgery. No studies have been published on effective plasma concentration (EC_50_) of meloxicam in cattle, whereas in horse and dog meloxicam EC_50_ was reported of approximately 0.2 and 0.36 μg/kg, respectively [54,55]. Although with obvious limitations, when considering these concentrations effective also for our buffaloes, they were maintained for more than two days in our study following IV administration of meloxicam at 0.5 mg/kg. However, it has to be further investigated, if NSAIDs effects are directly related to plasma drug concentrations and the real effective values in cattle and buffalo.

## Conclusion

The IV administration of meloxicam at 0.5 mg/kg b.w. in Mediterranean buffalo calves, shows encouraging effects represented by significant and prolonged analgesic effects, significant reduction of serum cortisol level as well as similar pharmacokinetic profile to bovine calves. Assessment of meloxicam effects and pharmacokinetics on a larger and different population, by different routes of administration and with other painful distresses or surgical procedures needs further scientific attention to fully understand its inclusion in the therapeutic approaches of these ruminants.

## Supporting information

S1 File Cagnardi et al. 2017. Raw data.
(XLSX)
